# Theranostic Gastrointestinal Endoscopy: Bringing Healing Light to the Lumen

**DOI:** 10.14309/ctg.0000000000000119

**Published:** 2020-03-04

**Authors:** Najib Nassani, Mira Alsheikh, Bobby Carroll, Diep Nguyen, Robert E. Carroll

**Affiliations:** 1 Division of Gastroenterology and Hepatology, Department of Medicine, University of Illinois at Chicago, Chicago, Illinois, USA;; 2 Department of Medicine, Zucker SOM at Hofstra/Northwell at Staten Island University Hospital, Staten Island, New York, USA;; 3 Department of Physics, Syracuse University, Syracuse, New York, USA;; 4 Department of Visual Information Processing, Institut de la Vision, Sorbonne Université, INSERM, CNRS, Paris, France.

## Abstract

Current conventional endoscopes have restricted the accuracy of treatment delivery and monitoring. Over the past decade, there have been major developments in nanotechnology and light triggered therapy, potentially allowing a better detection of challenging lesions and targeted treatment of malignancies in the gastrointestinal tract. Theranostics is a developing form of personalized medicine because it combines diagnosis and targeted treatment delivered in one step using advances in nanotechnology. This review describes the light-triggered therapies (including photodynamic, photothermal, and photoimmunotherapies), nanotechnological advances with nanopowder, nanostent, nanogels, and nanoparticles, enhancements brought to endoscopic ultrasound, in addition to experimental endoscopic techniques, combining both enhanced diagnoses and therapies, including a developed prototype of a “smart” multifunctional endoscope for localized colorectal cancer, near-infrared laser endoscope targeting the gastrointestinal stromal tumors, the concept of endocapsule for obscure gastrointestinal bleed, and a proof-of-concept therapeutic capsule using ultrasound-mediated targeted drug delivery. Hence, the following term has been proposed encompassing these technologies: “Theranostic gastrointestinal endoscopy.” Future efforts for integration of these technologies into clinical practice would be directed toward translational and clinical trials translating into a more personalized and interdisciplinary diagnosis and treatment, shorter procedural time, higher precision, higher cost-effectiveness, and less need for repetitive procedures.

## INTRODUCTION

Conventional gastrointestinal (GI) endoscopy allows the visualization of diseased regions and treatment at a macroscale. Lesions in the GI tract can be missed if they are localized in the submucosa, if their morphology is flat or depressed, if they are surrounded by an inflamed mucosa, or if they have not reached a detectable size (∼0.5–1 cm). In addition, conventional endoscopes do not allow dynamic monitoring while delivering treatment and can leave microscopic residual disease. “Theranostics” is considered a developing form of personalized medicine because it combines diagnosis and targeted treatment in one step using advances in nanotechnology while taking into account the uniqueness of disease signature.

Current endoscopes allow for sampling of lesions, removal of polyps, and control of bleed to the reachable parts of the GI tract. Newer techniques such as endoscopic submucosal dissection allow even “*en bloc* resection” of localized cancers of the GI tract. Endoscopic ablative therapies using thermal, photochemical, or radiofrequency energies applied to atypical lesions are other possibilities. However, current endoscopes limitations in both rigorously diagnosing and delivering targeted treatment arise mainly from the limited tip surface area that is small and does not allow integrating useful sensing and therapeutic features at a micrometric scale (1).

**Figure 1.**
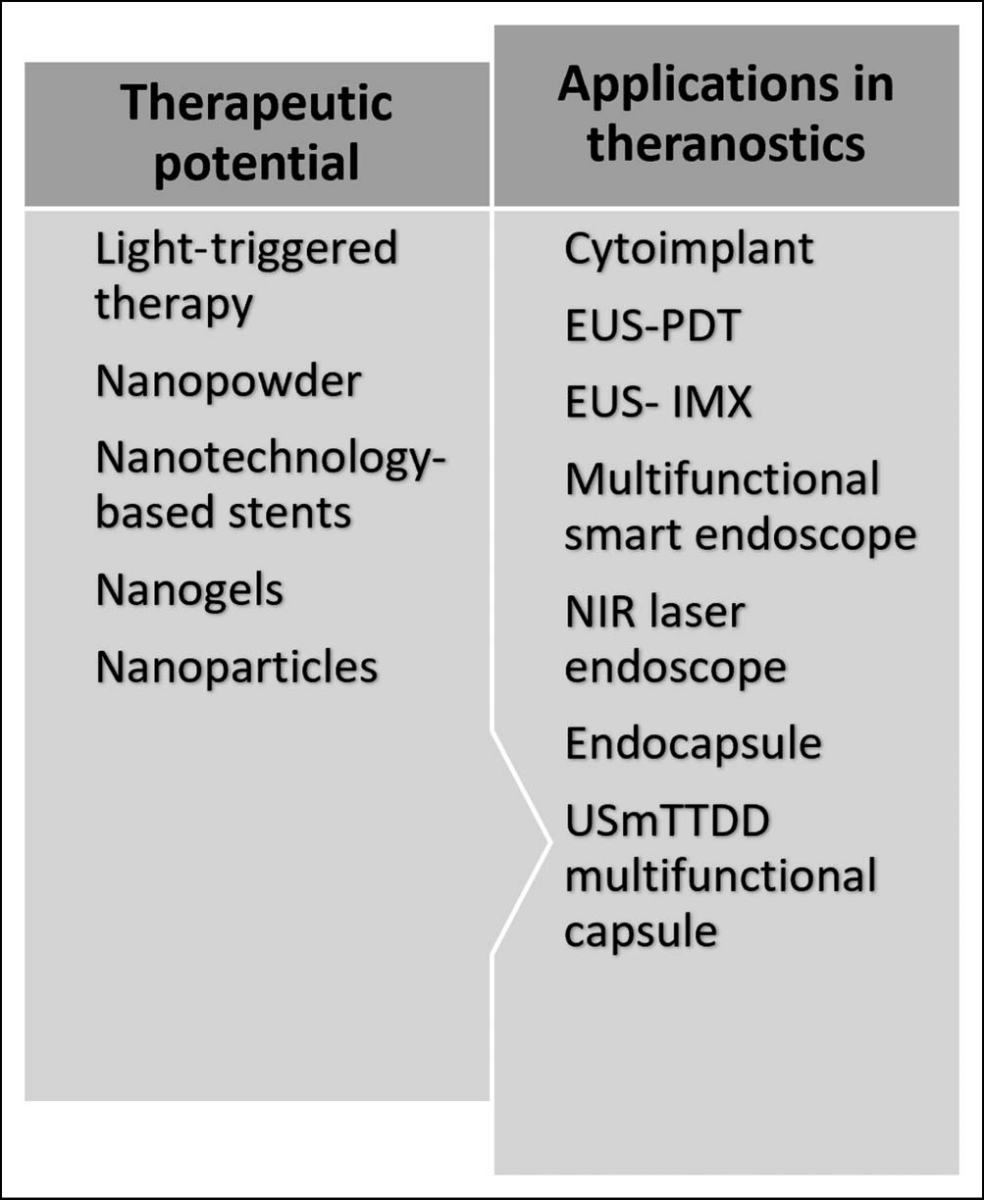
Summary of the described clinical and experimental techniques. EUS-PDT, endoscopic ultrasound-photodynamic therapy; EUS-IMX, endoscopic ultrasound-immunotherapy; NIR, near-infrared; USmTDD, ultrasound-mediated targeted drug delivery.

In this review, we present the main developing alternative strategies for clinical use to overcome the limitations of conventional endoscopy with a focus on light-triggered therapies, nanotechnological advances, and experimental endoscopic techniques combining both enhanced diagnoses and therapies, hence the term that we propose: “Theranostic gastrointestinal endoscopy.” Figure 1 summarizes the described clinical and experimental techniques in this review.

## THE THERAPEUTIC POTENTIAL

### Nanoparticles

**Figure 2.**
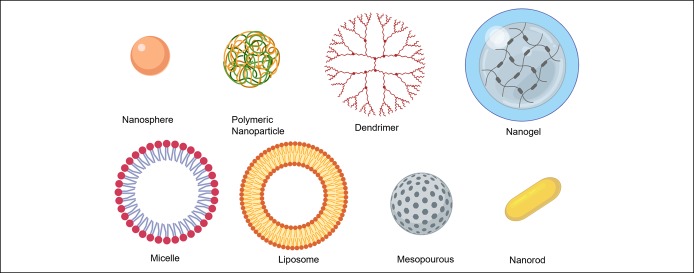
Schematic representation of different types of nanoparticles used as vehicles for treatment delivery.

Nanoparticles (NPs) are particles with at least one dimension that is measured in the nanometric scale (1 nm = 10^−9^ m) and up to 100 nm. NPs are attractive to the medical field given that biological and living systems are of the same order of magnitude at the cellular level. NPs have been studied as potential drug delivery vehicles owing to their advantageous size and surface chemistry tunability. NPs can be tailored to improve the drug delivery efficacy while simultaneously reducing unwanted accumulation in healthy cells. The inner shell of NPs can be loaded with therapeutic drugs while the outer shell can be functionalized with antibodies to target abnormal sites. Additional structural stability and reduced aggregation is possible by coating NPs' outer layer with polymers such as polyethylene glycol to modify their surface. Nanoparticles that have been studied for drug delivery include metals, semiconductors, both natural and synthetic polymer-based “soft NPs,” polymer superstructures called dendrimers, and lipids such as micelles and liposomes. Micelles and liposomes are 2 types of NPs with hydrophilic heads and hydrophobic tails, creating cores that may be loaded with a therapeutic agent or contrasting dyes. Both have controllable self-assembly and are useful for transporting hydrophobic drug agents in the cell's aqueous environment (2). Using an H22 cell model of colon cancer, the lipid NP shells loaded with Resveratol showed a faster mechanism of the drug (3). NPs composed of proteins and DNA can create virus complexes that can target macrophage-mediated diseases (4). Metallic nanoparticle cores can be synthesized in several shapes, including spheres, rods, tubes, and shells. Magnetic nanoparticles can be manipulated by magnetic fields and can be useful in chemotherapy (5) and for delivery of nucleic acids and proteins to a specific target site. In addition, nanoparticles whose surface is chemically functionalized with polymers, proteins, or liposomes can improve targeted drug administration. Hybrid gold-iron oxide NPs were synthesized using precursors of gold and iron oxide. These NPs were used as laser photothermal therapy (PTT) *in vitro* with selective destruction of colorectal cancer cells (6). Functionalization of these hybrid NPs was achieved using the A33scFv antibody and demonstrated a selectivity of 80% cellular uptake for human colorectal carcinoma SW1222 cells. The therapeutic effect of NPs is achieved via drug targeting, selective suppression of tumor growth, and potential magnetically induced hyperthermia. A schematic representation of different types of nanoparticles used as vehicles for treatment delivery is presented in Figure 2.

### Light-triggered therapy

**Figure 3.**
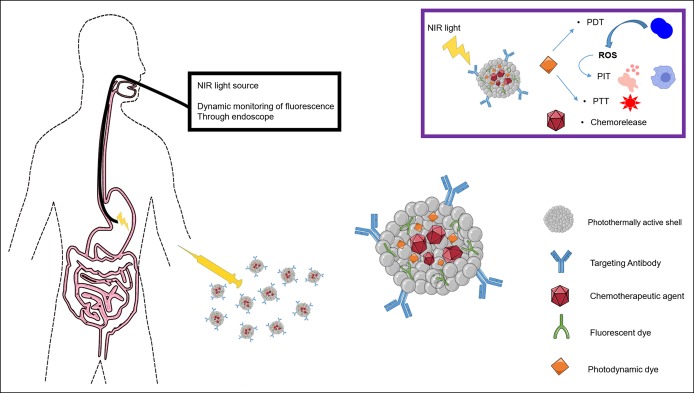
Scheme summarizing endoscopic use of light-triggered therapy. NIR, near-infrared; PDT, photodynamic therapy; PIT, photoimmunotherapy; ROS, reactive oxygen species.

Light-triggered therapy has been advanced as a promising cancer treatment option providing tunability and ease of irradiation focus which enables noninvasive and localized treatment (7). Laser irradiation of NPs affects cancerous cells through heat generation (PTT), reactive oxygen species (ROS) formation (photodynamic therapy [PDT]), photoimmunotherapy (PIT), or via local chemotherapeutic agent release. ROS are generated from the absorption of light into photosensitive molecules called photosensitizers present in the target cells. These photosensitizers absorb photons of a specific wavelength which cause them to be activated from a ground state to an excited state. As the photosensitizer returns to the ground state, it transfers energy to the adjacent available oxygen, producing a ROS that mediates local cellular toxicity. This singlet oxidation toxicity preferentially targets neoplastic cells but spares normal tissue because of the ROS's kinetic instability. PDT destroys the tumor cells also by damaging the tumor-associated vasculature, causing infarction, and by triggering an immune response against tumor cells (8). A scheme summarizing the endoscopic use of light-triggered therapy is presented in Figure 3.

PDT has been proposed as an alternative to esophagectomy in limited mucosal esophageal squamous cell carcinoma (9,10) and as a salvage therapy option for recurrent esophageal squamous cell carcinoma (11) and is also used for endoscopic ablation of high grade dysplasia in Barrett's esophagus (12). PDT is a palliative option for unresectable cholangiocarcinoma in association with biliary stent placement (13) because it was superior to the biliary stent alone in improving cholestasis and quality of life in addition to being well tolerated with minimal side effects.

### Nanopowder

Hemospray or TC-325 is an inorganic clay powder with no human- or animal-derived proteins (14) that works as a hemostatic agent by forming a gel barrier over the bleeding vessel and by enhancing clot formation (15) through an increase in the local concentration of clotting factors. Its effectiveness in achieving hemostasis has been shown in the setting of peptic ulcer bleeding (16), cancer-related upper GI hemorrhage (17), active variceal bleed (18), bleeding after therapeutic endoscopic procedures (19), and in lower GI bleed (20). It has been recently approved by the FDA for clinical use.

### Nanotechnology-based stents

Special nanocoatings were designed to optimize the biliary stent surface and avoid clogging by soil-release mechanism consisting in applying hydrophobic particles to coat the stents and hence reduce the sludge deposition. The chemical combination of the least hydrophobic silane and the most hydrophobic epoxide (500 g·mol^−1^) showed the best results. *In vitro* results are promising, but future studies are warranted (21).

### Nanogels

Nanogels or nanosize hydrogels are formed by the physical or covalent cross-linking of polymer chains into a polymer network. They have a large surface area to volume ratio along with controllable porosity, degradation, and surface functionalization (22), making them an exciting avenue for controlled drug release (23). Nanogels have been applied as novel oral drug delivery systems. When conjugated with 5-FU nucleoside floxuridine or with gemcitabine (24), they were reported to exert antitumor effect on drug-resistant tumors with permeability across the normally impermeable gastrointestinal barrier.

## INTEGRATION OF THERANOSTICS IN ENDOSCOPES

### Endoscopic ultrasound

Endoscopic ultrasound (EUS) is a well-established procedure for drainage of fluid collections in pancreatic pseudocysts, intraabdominal abscesses, loculated ascites (25,26), gallbladder drainage (27), celiac plexus neurolysis for pancreatic cancer–related pain (28), ethanol ablation of pancreatic insulinomas (29), and vascular interventions (30).

EUS became a central technique in the diagnosis and staging of GI and non-GI malignancies. It provides specimens for cytological and immunocytochemical assessments, gene expression patterns by polymerase chain reaction, microsatellite loss analysis and fluorescence *in situ* hybridization analysis (31), tissue architecture and morphology mainly by fine needle biopsy, molecular testing, and next-generation sequencing. Next-generation sequencing enables the study of the genome with the detection of pinpoint variations including point mutations and small insertions/deletions at reduced cost and turnaround time (31).

To increase the diagnostic yield, rapid on-site evaluation by a cytopathologist during the procedure is performed in some centers. It ensures that smears are adequately cellular and diagnostic, therefore theoretically reducing the number of needle passes performed and procedural time. Telepathology, where static histological or cellular images are acquired and sent to the remote pathologist who can also move the on-site microscope by using a robotically controlled microscope stage, is an alternative to the pathologist being on site to ensure that smears are diagnostic (32).

3D organoids can be created from the EUS-harvested tissue. These are models that mimic the tumor with its histologic features and temporal development helping in studying tumors, testing drugs, performing molecular and genetic interventions, and thus individualizing treatment (33,34).

Contrast-enhanced EUS (CE-EUS) quantifies the systemic circulation of different gastrointestinal organs. It uses inert gas in the microbubble in a phospholipid layer acting as a contrast agent (35). It is applied for the differentiation of pancreatic, gastrointestinal stromal tumor (GIST) and colorectal masses and aided in the prediction of tumor response to treatment against vascular angioneogenesis (36).

Most studies of the EUS for antitumor-agent delivery are still in phase I, confirming its safety awaiting further clinical trials. Cytoimplants consists of allogeneic-mixed lymphocyte culture delivered by the EUS-guided fine-needle injection in patients with advanced pancreatic carcinoma. Cytoimplant produces cytokines directly within the tumor inducing its regression (37) by activation of immune effector cells and by host antitumor effectors mechanisms. Adenovirus ONYX-015 is an engineered adenovirus with E1B 55 kDa gene deletion that replicates selectively in cells carrying the p53 mutation leading to targeted pancreatic adenocarcinoma cell death (38). A phase I/II trial of injecting ONYX-015 into unresectable pancreatic carcinoma via a transgastric route under EUS guidance was shown to be feasible and relatively well tolerated. EUS was studied as a mean of locally delivering PDT through optic fibers and radiofrequency. It was also used for placing variable treatment materials in the affected organs close to the gastrointestinal tract for brachytherapy. The feasibility and safety of delivering the EUS-guided PDT to the pancreas, liver, and spleen were proven in a porcine model. The photosensitizer “porfimer sodium” was injected intravenously 24 hours before applying the PDT with a 630 nm laser through a small diameter quartz optical fiber under EUS guidance. Remarkably, the extent and degree of necrosis induced by PDT were complete (100%) in the pancreas 2 days post-therapy (39).

Iodine 125 radioactive seeds were successfully implanted in the pancreatic tumors under EUS guidance, resulting in a moderate local tumor effect (40,41). EUS has been implicated in immune therapies by administering TNFerade, an immunotherapeutic agent that exhibits its effect through transporting human tumor necrosis factor alpha gene to the cancer cells using replication-deficient adenovirus that has a tumor necrosis factor alpha complementary DNA ligated to the Egr-1 radiation-inducible promoter gene integrated into its genome (42). When combined with chemoradiotherapy, TNFerade was associated with prolonged survival in esophageal cancer (43). Oncogel/paclitaxel (44) or gemcitabine (45) intratumoral administration and EUS-guided immunotherapy (46,47) have also been described.

### Multifunctional theranostic endoscope-based interventional system

A “multifunctional endoscope-based interventional system” or “smart” endoscope has been proposed as a “closed-loop theranostic solution for colon cancer.” Synergistic effects on enhancing the tumor detection accuracy and providing treatment were exhibited by the combination of transparent electronics that allows visualization throughout the camera (fluorescence mapping and/or phototherapies), tissue characterization (through impedance and pH sensing), and removal of colon cancer (radiofrequency ablation therapy), whereas custom-designed biocompatible nanoparticles were loaded with phototherapeutic and chemotherapeutic agents activated with light (1).

Active targeting of NPs was achieved through conjugating cetuximab-specific antibody, targeting the epidermal growth factor receptors that are overexpressed in the HT-29 colon cancer cells on the surface of NPs. NPs had a photothermally active core-shell structure that was loaded with fluorescence dye (rhodamine B), photodynamic dyes PDT (chlorin e6), and chemodrugs (doxorubicin; Dox). After IV injection of the NPs, laser light emitted through the endoscope accessed suspicious sites targeted by NPs and observed through the transparent bioelectronics on the endoscope camera. HT-29 cancer cells were distinguished from the normal cells through impedance and pH differences. The cancerous tissue observed was resected using the forceps followed by radiofrequency ablation. Continuous monitoring of temperature, contact, and cell/tissue viability were ensured by the transparent bioelectronics. The photoactivation of NPs was localized to laser-irradiated regions and controlled by modulating the laser wavelength. NPs were locally activated using a continuous-wave red laser to induce PDT (ROS) and by using near-infrared (NIR) lasers for PTT (heat) and chemodrug release (Dox).

This “smart” endoscope has been tested *in vitro*, *ex-vivo*, and *in vivo* in the BALB/c nude mouse model. The most accentuated decrease in the tumor volume was in the combined therapy group (PDT, PTT, and chemotherapy) and was mediated by apoptosis.

### NIR laser endoscope targeting GISTs

Fujimoto et al. (48) used the property of NIR light of penetrating tissues to develop a therapeutic strategy, addressing GISTs that are submucosal. NIR light at wavelengths of 600–700 nm remains at a depth of 5–20 mm corresponding to the level of these submucosal lesions (49). c-KIT markers on the GIST-T1 cells were targeted by anti c-KIT antibody (the murine: 12A8) conjugated to a photosensitizer (IR700). Fluorescence colonoscopy was achieved through autofluorescence imaging endoscopy. This strategy of combining a photosensitizer with an antibody against a tumor-specific molecule and irradiating the affected region visualized by fluorescence leads to PIT. The mechanism of the GIST cell death using this technique appears to be related to acute necrosis and late apoptosis induced by accumulation of ROS. This approach is tumor specific, effective, and tunable because imaging guidance is achieved with minimal irradiation, whereas irradiating more strongly would allow treatment. The tumor volume in mice treated with IR700-12A8-mediated PIT was significantly decreased by 35% soon after treatment in all mice, and the tumors disappeared completely in 4 of 7 mice thereafter. The limitations for this technique reaching a clinical stage are mainly studying the humanized antibody because 12A8 is a murine anti-c-KIT antibody and developing NIR fluorescence endoscopes ready for clinical use (48).

### Endocapsule for obscure GI bleed

The concept of a theranostic capsule loaded with nanoparticles designed to target the bleeding site in the GI tract through endothelin-1 then mark these sites with fluorescein dye and release the treating agent (fibrin) to ensure hemostasis at the bleeding site has been proposed. One of the hypothetically described methods relied on the autologous fibrin glue and autologous platelet-rich fibrin derived from the patient's own blood (50).

### Theranostic ultrasound-mediated targeted drug delivery (USmTDD) multifunctional capsule

A proof-of-concept therapeutic capsule using ultrasound-mediated targeted drug delivery (USmTDD) with fluorescence imaging guidance has been conceived (51). This proof-of-concept capsule combines videocapsule endoscopy with microultrasound fluorescence imaging. It aims at a targeted treatment for localized diseases in the GI tract. First, a capsule with microultrasound allows sensing and marking of the diseased areas of the GI tract by fluorescent particles. Then, a therapeutic dedicated capsule allows subsequent treatment of the insonated areas. Insonation causes a decrease in the cellular barrier function and thus permeability to drugs (52,53). USmTDD has not reached clinical use and is limited to a proof-of-concept theranostic capsule endoscopy device: SonoCAIT. The specific challenge is the miniaturization of the focused-US transducer (54).

Other potential therapeutic uses of endoscopic capsules include mounting of the nitinol clip released by an external magnetic signal focused on the capsule equipped with 4 permanent magnets (55) and obtaining biopsies using rotational razor mechanism, microspikes, or 2 cylindrical razors (56–58).

## CONCLUSION

The past decade has been marked by significant endoscopic advances mainly in refining visualization and endoscopic dissection and ablative techniques. These endoscopic advances were coupled with developing and functionalizing new nanoparticles.

Using endoscopy to combine diagnosis with the delivery of a targeted treatment can be termed “Theranostic gastrointestinal endoscopy,” another potential added tool to the therapeutic arsenal of delivering a personalized care targeting the molecular signature of diseases and their hosts. Challenges for clinical application of these technologies are mainly related to innovating new endoscopes that allow integrating more complex functions and to further studying the behavior of light-triggered nanoparticles in humans.

Future efforts would be directed toward translational and clinical trials with multifunctional endoscopes and capsules, with the integration of the rising field of artificial intelligence. This multifunctionality could clinically translate into a more personalized and interdisciplinary treatment, shorter procedural time, higher precision, higher cost-effectiveness, and less need for repetitive procedures.

## CONFLICTS OF INTEREST

**Guarantor of the article:** Najib Nassani, MD, MSc.

**Specific author contributions:** N.N.: study idea and concept, literature review, drafting the manuscript, reviewing the manuscript, and finalizing the manuscript. M.A.: literature review, drafting the manuscript, and reviewing the manuscript. B.C.: literature review, drafting the manuscript, and reviewing the manuscript. D.N.: literature review, drafting the manuscript, and reviewing the manuscript. R.E.C.: literature review, drafting the manuscript, reviewing the manuscript, and finalizing the manuscript. All authors agreed on the submitted version of the manuscript.

**Financial support:** None to report.

**Potential competing interests:** None to report.
